# Erythrocyte Selenium as a Potential Key Indicator for Selenium Supplementation in Low-Selenium Populations: A Selenium Supplementation Study Based on Wistar Rats

**DOI:** 10.3390/nu16223797

**Published:** 2024-11-05

**Authors:** Cunqi Lv, Ruixiang Wang, Qingyu Zeng, Chen Feng, Guijin Li, Shuxiu Hao, Jiacheng Li, Cheng Wang, Huixin Sun, Linlin Du, Yu Zhang, Xinshu Wang, Tong Wang, Qi Li

**Affiliations:** 1 Institute of Keshan Disease, Chinese Center for Endemic Disease Control, Harbin Medical University, Harbin 150081, China; 2022020106@hrbmu.edu.cn (C.L.); 2020020149@hrbmu.edu.cn (R.W.);; 2 Key Laboratory of Etiology and Epidemiology, Education Bureau of Heilongjiang Province (23618504) & Ministry of Health, Harbin Medical University, Harbin 150081, China; 3 Department of Clinical Medicine, Queen Mary College, Nanchang University, Nanchang 330038, China; 4 Department of Radiotherapy, Harbin Medical University Cancer Hospital, Harbin 150081, China

**Keywords:** nutritional status, erythrocyte selenium, serum selenium, hair selenium, glutathione peroxidase

## Abstract

Background: Selenium (Se) is an essential trace element for maintaining human health, with significant antioxidant and immunoregulatory functions. Inadequate Se intake may be associated with Keshan disease, Kashin–Beck disease, and hypothyroidism. However, effective indicators for scientifically guiding Se supplementation in Se-deficient populations are still lacking. Objectives: This study aims to explore the dynamic distribution of Se across various nutritional biomarkers and major organs in rats through a Se supplementation experiment, as well as the pairwise correlations between them, in order to identify reliable nutritional indicators for evaluating Se levels in the body. Methods: Se levels in hair, blood, and major tissues and organs were determined by atomic fluorescence spectrometry, and glutathione peroxidase (GSH-Px) levels were measured using an ELISA. Results: Se supplementation significantly increased Se levels in rat blood, hair, and major organs, as well as GSH-Px levels in blood. Se primarily accumulated in the liver and kidneys, followed by myocardium, spleen, and muscles. Serum and plasma Se were found to be the best indicators of short-term Se intake, while erythrocyte Se levels showed a stronger correlation with Se levels in tissues and organs, making it a better marker for assessing long-term Se nutritional status compared to hair Se. Conclusions: This study demonstrates the potential of erythrocyte Se levels as an indicator for evaluating long-term Se nutritional status, providing scientific evidence for Se nutritional assessments.

## 1. Introduction

Selenium (Se) is an essential trace element for humans, playing a variety of critical physiological roles, including antioxidant, immunoregulatory, anti-inflammatory, anticancer, and anti-infection functions [1]. When Se concentrations are too low, its biological functions cannot be fully exerted [2], potentially increasing the risk of Keshan disease [3], Kashin–Beck disease, hypothyroidism, and miscarriage and other reproductive and obstetric complications [4,5]. Conversely, excessive Se intake may induce oxidative stress, leading to hair loss, nail deformities, neurological symptoms, and even life-threatening conditions [6,7,8].

Se nutritional status is commonly assessed by measuring Se levels in hair, plasma, and serum [9]. Hair Se levels are widely used to assess long-term Se intake due to the ease of sample collection, storage, transport, and high compliance [10]. Serum or plasma Se levels mainly reflect recent dietary Se intake [11]. Glutathione peroxidase (GSH-Px), known for its significant antioxidant properties, exhibits decreased activity during Se deficiency but recovers rapidly after Se supplementation. This makes it a common indicator of Se nutritional status and the body’s antioxidant capacity [12].

Our previous study, which measured hair Se levels in 3026 residents across 28 provinces in China, found that 40.9% of the participants exhibited varying degrees of Se deficiency [10]. Dietary interventions and supplementation in Se-deficient areas have proven to be effective preventive measures [13]. However, given the narrow safety range of Se intake, uncontrolled long-term supplementation may lead to Se excess, posing potential health risks [14,15]. Due to economic constraints, sample preservation, transport, and technical limitations, it is challenging to comprehensively assess all Se nutritional indicators in practice. Therefore, identifying an efficient, stable, and representative biomarker to guide Se supplementation strategies is imperative.

Given this background, we conducted a Se supplementation experiment in Wistar rats to further explore the effects of Se supplementation on Se nutritional biomarkers (hair, whole blood, serum, plasma, erythrocyte, and GSH-Px), as well as Se levels in major tissues and organs (liver, kidneys, spleen, myocardium, and muscles). This study aims to explore the dynamic distribution of Se across various nutritional biomarkers and major organs in rats through a Se supplementation experiment, as well as the pairwise correlations between them, in order to identify reliable nutritional indicators for evaluating Se levels in the body.

## 2. Materials and Methods

### 2.1. Chemicals

Sodium selenite (SS) was purchased from Sigma-Aldrich (St. Louis, MO, USA).

### 2.2. Dosage Calculation

According to the guidelines for nutritional requirements, the recommended nutrient intake (RNI) for Se in humans is 55 µg/day, and the tolerable upper intake level (UL) is 400 µg/day [16]. To ensure the efficacy of the experiment without causing Se toxicity in rats, a human Se intake of 200 µg/day was used as the basis, assuming an average adult weight of 60 kg and applying a conversion factor (6.25) to account for animal tolerance [17]. The final dosage of SS for the rats was calculated to be 200 µg/L. Thus, a 200 µg/L SS solution was used for Se supplementation in the drinking water of the experimental group.

### 2.3. Selenium Supplementation Experiment in Rats

#### 2.3.1. Animal Grouping and Sample Size

We strictly adhered to the 3R principles in determining the number of rats used in this study. The experiment is divided into five time phases, with sampling conducted once every seven days. For the control group, the minimum number of animals was maintained at five per stage, while in the selenium-supplemented (SS) group, the number was increased to 10 per stage to account for potential individual variability following supplementation. To prevent any impact from unforeseen losses, an additional 5 rats were included for the control group and 10 for the SS group. Additionally, to evaluate whether selenium levels in the control group changed significantly during the experiment compared to pre-experiment levels, a baseline group of 10 rats was established. Thus, the final composition included 10 rats in the baseline group, 30 in the control group, and 60 in the SS group, totaling 100 rats.

#### 2.3.2. Animal Husbandry, Grouping, and Sampling Protocol for Selenium Supplementation Study in Wistar Rats

A total of 100 male Wistar rats, aged 6–8 weeks, were purchased from Beijing Vital River Laboratory Animal Technology Co., Ltd. (Beijing, China) and housed at the SPF-level Transgenic Animal Research Center of Harbin Medical University. The rats were kept in a 12 h light/dark cycle with free access to food and water. After a one-week acclimatization period, the rats were randomly divided into a baseline group (*n* = 10), an SS group (*n* = 60), and a control group (*n* = 30). The baseline group was sacrificed immediately, while the SS and control groups underwent a 5-week intervention. The SS group received drinking water supplemented with 200 µg/L SS (Se: 91.32 µg/L), while the control group received regular water (Se: 0 µg/L). There was no difference in the feed provided to the two groups of rats (Se: 0.15 mg/kg). Sampling points were taken every 7 days. In the first 4 weeks, 10 rats from the SS group and 5 from the control group were randomly selected and euthanized weekly. The remaining rats were sacrificed in the fifth week. Blood, hair, myocardium, liver, kidneys, spleen, and thigh muscles (the quadriceps muscles) were collected post-mortem. Hair samples (from the dorsum and ventral areas) were stored in polyethylene ziplock bags and kept in a dry, dark environment. Myocardium, liver, kidneys, spleen, and thigh muscles were weighed and stored along with whole blood, plasma, and serum samples in an ultra-low temperature freezer at −80 °C.

Blinding was applied throughout the processes of group assignment, housing, euthanasia, and relevant indicator assessments to ensure that the experimenters were unaware of the grouping conditions. The study strictly adhered to animal ethical standards and was approved by the Ethics Committee of the Center for Endemic Disease Control, Harbin Medical University (Approval No. hrbmuecdc20220402).

### 2.4. Organ Coefficient

The spleen, liver, kidney, and heart of the rats were excised and weighed. The relative weight of each organ was calculated based on the final body weight measured on the day of organ collection. The organ coefficients were calculated as follows:

Organ coefficient (g/100 g) = organ weight/rat body weight × 100.

### 2.5. Measurement of Selenium Levels

Se levels were determined using an atomic fluorescence spectrometer (AFS 933, Beijing Titan Instrument Co., Beijing, China).

#### 2.5.1. Sample Preparation

(1) Hair: 0.1 g of clean, dry hair samples were placed into 50 mL digestion vessels.

(2) Serum, plasma, and whole blood: 100 µL of each sample was placed into 50 mL conical flasks.

(3) Erythrocytes: Approximately 0.1 g of erythrocytes were weighed and placed into 50 mL conical flasks.

(4) Organs: Frozen myocardium, liver, kidneys, spleen, and thigh muscles were cut into small pieces, and approximately 0.1 g of each sample was placed into 50 mL conical flasks.

(5) Standard materials: Approximately 0.1 g of human hair reference material was weighed and placed into 50 mL conical flasks.

#### 2.5.2. Determination of Selenium Levels in Samples

A mixture of 5 mL of acid (nitric acid and perchloric acid in a 4:1 ratio) was added to digest the samples or standard materials, which were left to stand overnight. The next day, thermal digestion was carried out at 180 °C using an electric sand bath. After cooling to room temperature, 5 mL of a hydrochloric acid solution (1:1 ratio of concentrated hydrochloric acid and deionized water) was added for reduction. Then, 1 mL of potassium ferricyanide solution was added, and the samples were diluted to 10 mL with 5% hydrochloric acid. Se levels were measured using the AFS-933 dual-channel atomic fluorescence spectrometer, and a standard curve was drawn based on Se content and fluorescence values. The blank samples, human hair reference material, and sample fluorescence values were measured sequentially. The Se content was determined using the standard curve. The recovery rate of Se was 88.8–117.1%.

### 2.6. Measurement of Glutathione Peroxidase (GSH-Px) Levels

GSH-Px levels in serum or plasma were measured using a GSH-Px assay kit (Jiancheng Biotech, Nanjing, China). Serum or plasma samples were diluted with physiological saline, and whole blood samples were prepared into hemolysates. Pre-experiments were conducted to determine the optimal dilution factor, ensuring an inhibition rate between 45% and 50%. Reagents were prepared according to the kit instructions, and GSH solutions and test samples were added to enzyme and non-enzyme tubes. The reaction was conducted in a 37 °C water bath. After the reaction, the supernatant was collected by centrifugation, and the samples were added to a microplate. Optical density (OD) values were measured at 405 nm using a microplate reader (BioTek Instruments, Winooski Charlotte, VT, USA).

### 2.7. Data Analysis

Data were analyzed using SPSS 17.0. The normality of the data was assessed using the Shapiro–Wilk test. For data that followed a normal or approximately normal distribution, the results were expressed as the mean ± standard deviation (SD), and group differences were analyzed using a *t*-test of independent samples. Spearman’s rank correlation analysis was performed, with correlation coefficients represented by *r_s_*. A *p*-value of <0.05 was considered statistically significant.

## 3. Results

### 3.1. Dietary Intake, Body Weight, and Organ Coefficients of Rats at Various Time Points

**Figure 1:**
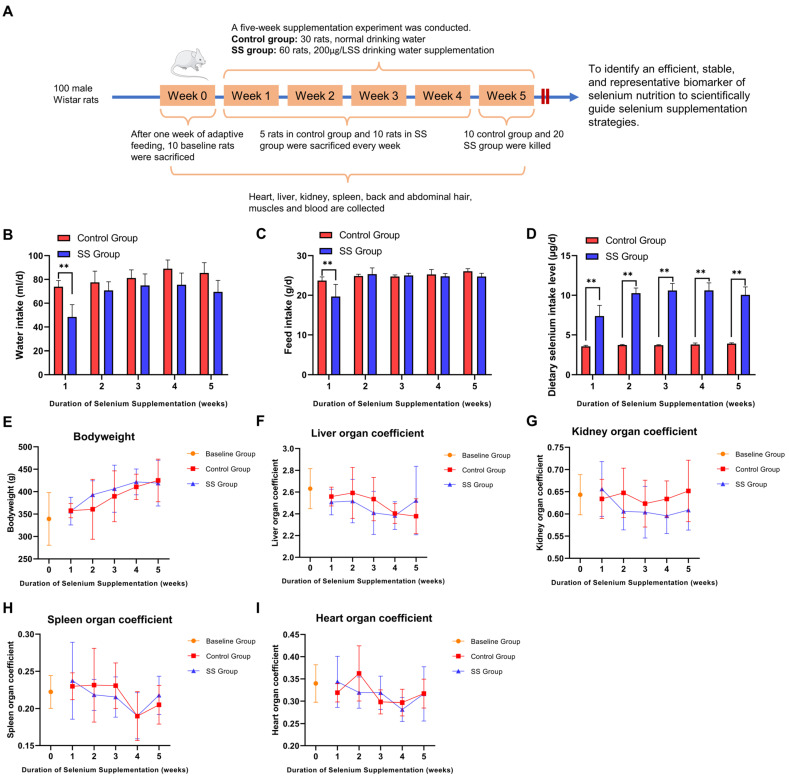
Dietary intake, body weight, and organ coefficients of rats in the control group and the Sodium selenite (SS) group at different time points. Flowchart of the selenium (Se) supplementation experiment in rats (**A**). Changes in water intake (**B**), feed intake (**C**), Se intake from diet (**D**), body weight (**E**), liver organ coefficient (**F**), kidney organ coefficient (**G**), spleen organ coefficient (**H**), and heart organ coefficient (**I**) at different time points. Asterisk indicates statistically significant differences between the SS group and the control group at corresponding time points, ** *p* < 0.01.

To identify the optimal indicator of Se nutritional status, we conducted a Se supplementation experiment in rats (Figure 1A). As shown in Figure 1B,C, the water intake and feed consumption levels of the rats were not significantly different between the control group and the SS group from the second to fifth week (*p* > 0.05), except in the first week, where the control group had a significantly higher intake than the SS group (*p* < 0.01). Further evaluation of the total Se intake from the diet (including feed and water), as shown in Figure 1D, revealed that the Se intake level in the SS group was significantly higher than that of the control group throughout the supplementation period (*p* < 0.01). The organ coefficients were assessed as shown in Figure 1E–I, and no significant differences in body weight or organ coefficients (liver, kidneys, spleen, heart) were observed between the SS rats and the control group at any time point during the supplementation period (*p* > 0.05). This suggests that Se supplementation had no significant impact on the body weight or the major organs of the rats.

### 3.2. Selenium Nutritional Indicators in Rats at Various Time Points

**Figure 2:**
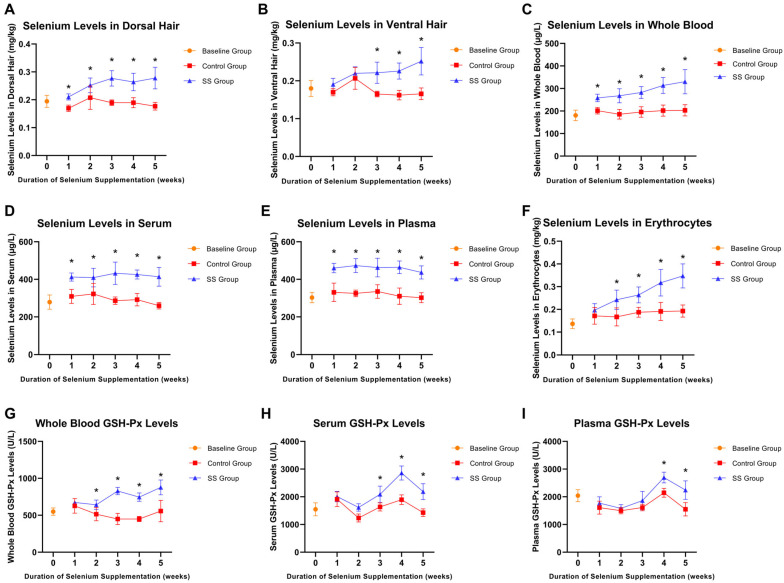
Changes in Se nutritional biomarkers in rats from the control group and SS group at different time points. Changes in dorsal hair Se (**A**), ventral hair Se (**B**), whole blood Se (**C**), serum Se (**D**), plasma Se (**E**), erythrocyte Se (**F**), whole blood GSH-Px (**G**), serum GSH-Px (**H**), and plasma GSH-Px (**I**) levels at different time points in rats. Asterisk indicates statistically significant differences in Se levels and GSH-Px activity between the SS group and the control group at corresponding time points, * *p* < 0.05.

To further explore the effect of Se supplementation on Se nutritional indicators in rats, we measured the Se levels in dorsal hair, ventral hair, whole blood, serum, plasma, and erythrocytes, along with GSH-Px levels in serum, plasma, and whole blood. As shown in Figure 2, the Se nutritional indicators in the control group rats did not exhibit significant fluctuations throughout the experimental period. In contrast, the Se nutritional indicators in the SS supplementation group showed varying degrees of increase compared to the control group (*p* < 0.05). Specifically, Se levels in dorsal hair, ventral hair, whole blood, and erythrocytes showed a continuous increase throughout the supplementation (Figure 2A–C,F). In contrast, serum and plasma Se levels increased sharply during the first week but plateaued over the subsequent weeks (Figure 2D,E). Additionally, GSH-Px levels in the serum, plasma, and whole blood samples showed a significant increase (*p* < 0.05). As the supplementation period progressed, GSH-Px levels continued rising; however, after the fourth week of supplementation, the increase was no longer statistically significant compared to the control group (Figure 2G–I).

### 3.3. Selenium Levels in Major Tissues and Organs of Rats at Various Time Points

**Figure 3:**
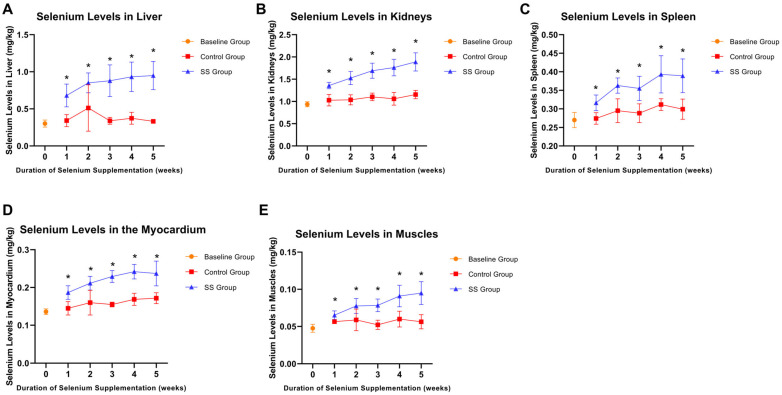
Changes in Se levels in various tissues and organs of rats from the control group and SS group at different time points. The Se level changes in liver (**A**), kidneys (**B**), spleen (**C**), myocardium (**D**), and muscles (**E**) at different time points in rats. Asterisk indicates statistically significant differences in Se levels between the SS group and the control group at the corresponding time points, * *p* < 0.05.

To validate the impact of Se supplementation on the Se concentrations in major tissues and organs, we measured Se concentrations in the liver, kidneys, spleen, myocardium, and muscles. As shown in Figure 3, consistent with the Se nutritional indicators, the Se levels in the major tissues and organs of the control group did not exhibit significant changes. In contrast, the Se content in these organs significantly increased in the SS supplementation group, with a continuous upward trend as the supplementation period progressed.

### 3.4. Selenium Enrichment in Nutritional Indicators and Major Organs

To assess Se accumulation in major organs, we evaluated the Se levels in the nutritional indicators and organs of rats after five weeks of supplementation, comparing them with the control group. As shown in Table 1, in the control group, Se was primarily concentrated in the kidneys, followed by the liver and spleen, with the lowest levels in the muscles. After five weeks of supplementation, while the kidneys remained the organ with the highest Se concentration, the liver showed the greatest fold change in Se accumulation (2.87), followed by the muscles (1.69) and the kidneys (1.63), with the smallest change observed in the spleen (1.30). Among the Se nutritional indicators, erythrocytes exhibited the highest fold change in Se accumulation (1.80), followed by whole blood Se (1.63) and serum Se (1.59).

### 3.5. Correlation Analysis Between Selenium Nutritional Indicators and Major Organs

To explore the correlation between Se nutritional indicators and Se levels in major organs, we performed pairwise correlation analyses. As shown in Table 2, Se levels in the myocardium, liver, kidneys, spleen, and muscles exhibited strong intercorrelations with each other (*r_s_* > 0.8). Further analysis revealed that whole blood and erythrocyte Se concentrations were highly correlated with Se levels in these organs (*r_s_* > 0.8). In contrast, hair Se levels showed weaker correlations with organ Se levels, with correlation coefficients mostly ranging from 0.7 to 0.8. Additionally, GSH-Px levels in whole blood showed higher correlations with Se levels in the liver, kidneys, spleen, myocardium, and muscles (0.6 < *r_s_* < 0.8) compared to serum and plasma Se levels (0.3 < *r_s_* < 0.7).

## 4. Discussion

In our previous cross-sectional study on Se nutritional levels in the Chinese population, we found that 40.9% of residents exhibited varying degrees of Se deficiency, with 5.3% experiencing severe deficiency [10]. Environmental factors and dietary habits are the primary determinants of human Se intake [18,19]. In China, insufficient dietary Se intake remains a significant challenge [8,20], particularly in low-Se regions, where Se deficiency disrupts the food chain and poses serious health risks to the population [21,22]. Research indicates that over 50% of Chinese children and 80% of elderly individuals consume dietary Se below the estimated average requirement level (EAR), highlighting the urgent need for Se supplementation in low-Se areas [23,24]. Therefore, a reliable Se nutritional indicator is needed to guide effective supplementation strategies.

As demonstrated in Figure 2 and Figure 3, Se supplementation in rats significantly elevated Se levels in the liver, kidneys, spleen, myocardium, muscles, blood, and hair, while also enhancing GSH-Px activity. Serum and plasma Se levels reached saturation within the first week of supplementation, whereas Se levels in the hair, whole blood, erythrocytes, and major organs continued rising with extended supplementation. Serum and plasma Se levels remain important indicators for assessing short-term Se nutritional status, while erythrocyte and hair Se levels are more suitable for evaluating long-term Se nutritional status. Whole blood Se, which includes both plasma and erythrocyte Se, can comprehensively assess both long-term Se nutritional levels and short-term Se intake. These findings are consistent with the study by Solé-Navais et al. in Norway, which similarly concluded that whole blood Se concentration reflects both long-term status and short-term Se intake, while plasma/serum Se reflects only short-term status [25]. Moreover, studies by Costa and Stefanowicz confirmed that erythrocyte Se is less influenced by external factors, due to the longer lifespan of erythrocytes, making it a more accurate reflection of long-term Se nutritional status [26,27]. Although hair Se is often used to assess long-term nutritional status [3,28,29], its susceptibility to environmental factors and the potential reluctance of participants (e.g., women) to provide hair samples limit its utility as an indicator. GSH-Px, a Se-dependent antioxidant enzyme, plays a vital role in reducing oxidative stress and maintaining cellular redox balance, serving as a direct indicator of the body’s antioxidant capacity [30,31]. In this study, Se supplementation significantly increased GSH-Px levels in rat blood, indicating an improvement in Se nutritional status. However, after four weeks of supplementation, GSH-Px levels plateaued, suggesting that the body had reached an optimal Se level, entering a state of Se nutritional balance. These results indicated that GSH-Px may serve as a useful biomarker for guiding Se supplementation strategies.

Our study also found that after five weeks of Se supplementation, Se levels in the liver and kidneys increased significantly, while the spleen and myocardium showed more moderate increases. The preferential accumulation of Se in the liver and kidneys is closely related to their roles in Se metabolism, storage, and excretion [32,33,34]. The liver is the primary site of selenoprotein synthesis, particularly for antioxidant enzymes like GSH-Px, which helps cells resist oxidative damage. The liver also distributes Se throughout the body in biologically active forms (e.g., SELENOP) [35]. Kidneys excrete excess Se, eliminating it in inorganic forms or Se metabolites via urine [36,37]. The relatively smaller changes in myocardial Se levels may be due to the myocardium’s sensitivity to Se deficiency. Numerous studies have demonstrated a close association between selenium deficiency and cardiovascular diseases, with selenium deficiency increasing the risk of Keshan disease (endemic cardiomyopathy) [38], heart failure [39], and other cardiac disorders [40]. Liver and kidney function should be given close attention in future human Se supplementation studies to mitigate potential risks of damage.

Furthermore, Se levels show a high correlation among various organs and tissues. Among Se nutritional indicators, erythrocyte Se and whole blood Se had the highest correlation with Se levels in organs, followed by hair Se. Serum and plasma Se showed the weakest correlation, possibly because they reached saturation early in the supplementation process. In contrast, hair Se, due to its growth characteristics, may lag behind actual Se intake changes and be more influenced by environmental factors. Therefore, erythrocyte Se, compared to hair Se, is a more reliable indicator for evaluating long-term Se nutritional status. These findings provide scientific evidence for guiding future Se supplementation strategies in Se-deficient areas.

This study has several limitations. First, the exclusive use of male rats may introduce selection bias, as sex differences could influence the results. Additionally, we chose a 2:1 ratio of Se-supplemented rats to control rats to more comprehensively assess the effects of Se supplementation, rather than the traditional 1:1 ratio. Future research should validate the efficacy of erythrocyte Se as an indicator of long-term Se nutritional status using both male and female rats and more balanced group ratios to enhance the generalizability and applicability of the findings. Furthermore, this study only measured GSH-Px levels in whole blood, serum, and plasma, without evaluating GSH-Px levels in erythrocyte or other biomarkers reflecting the body’s antioxidant capacity. In future studies, these additional parameters will be assessed to provide a more comprehensive evaluation of selenium’s impact on the body’s antioxidant function.

## 5. Conclusions

This study demonstrates that Se supplementation significantly increases Se levels in rat blood, hair, GSH-Px, liver, kidneys, spleen, myocardium, and muscles. Plasma and serum Se levels are important indicators for assessing short-term Se nutritional status, while erythrocyte Se is more effective for evaluating long-term Se nutritional status and proves to be more reliable than hair Se. The liver and kidneys are the primary sites of Se accumulation, both in the absence of and following supplementation. These findings provide critical scientific evidence to guide future Se supplementation strategies and optimize Se nutrition in deficient populations.

## Figures and Tables

**Table 1:** Se concentrations of biomarkers in rats from the control and SS groups.

Selenium Biomarkers	Control Group	SS Group	*t*-Value	*p*-Value	SS Group/ Control Group
Myocardial Selenium (mg/kg)	0.17	0.24	6.00	<0.0001	1.38
Muscle Selenium (mg/kg)	0.06	0.09	7.26	<0.0001	1.69
Spleen Selenium (mg/kg)	0.30	0.39	5.72	<0.0001	1.30
Liver Selenium (mg/kg)	0.33	0.95	10.16	<0.0001	2.87
Kidney Selenium (mg/kg)	1.16	1.89	10.75	<0.0001	1.63
Dorsal Hair Selenium (mg/kg)	0.18	0.28	7.92	<0.0001	1.57
Ventral Hair Selenium (mg/kg)	0.17	0.25	7.13	<0.0001	1.52
Whole Blood Selenium (µg/L)	203.08	330.41	6.74	<0.0001	1.63
Erythrocyte Selenium (mg/kg)	0.19	0.35	8.25	<0.0001	1.80
Plasma Selenium (µg/L)	302.48	436.65	10.63	<0.0001	1.44
Serum Selenium (µg/L)	259.85	413.92	8.89	<0.0001	1.59
Whole Blood GSH-Px (U/L)	556.34	877.06	6.97	<0.0001	1.58
Plasma GSH-Px (U/L)	1549.51	2243.05	5.83	<0.0001	1.45
Serum GSH-Px (U/L)	1427.55	2183.52	7.48	<0.0001	1.53

**Table 2:** Correlation analysis of Se indicators in rats.

	Myocardial Se	Muscle Se	Spleen Se	Whole Blood Se	Liver Se	Kidney Se	Erythrocyte Se	Dorsal Hair Se	Ventral Hair Se	Plasma Se	Serum Se	Whole Blood GSH-Px	Plasma GSH-Px	Serum GSH-Px
Myocardial Se	1	0.877 **	0.834 **	0.843 **	0.868 **	0.890 **	0.838 **	0.816 **	0.721 **	0.706 **	0.726 **	0.741 **	0.415 **	0.602 **
Muscle Se		1	0.854 **	0.834 **	0.862 **	0.859 **	0.855 **	0.778 **	0.771 **	0.614 **	0.636 **	0.735 **	0.455 **	0.581 **
Spleen Se			1	0.779 **	0.835 **	0.810 **	0.830 **	0.732 **	0.718 **	0.613 **	0.606 **	0.688 **	0.436 **	0.536 **
Whole Blood Se				1	0.799 **	0.888 **	0.887 **	0.764 **	0.727 **	0.759 **	0.765 **	0.764 **	0.490 **	0.689 **
Liver Se					1	0.859 **	0.806 **	0.789 **	0.711 **	0.723 **	0.668 **	0.722 **	0.397 **	0.571 **
Kidney Se						1	0.897 **	0.803 **	0.716 **	0.696 **	0.706 **	0.788 **	0.405 **	0.623 **
Erythrocyte Se							1	0.738 **	0.708 **	0.612 **	0.634 **	0.736 **	0.486 **	0.614 **
Dorsal Hair Se								1	0.794 **	0.707 **	0.673 **	0.680 **	0.378 **	0.481 **
Ventral Hair Se									1	0.566 **	0.593 **	0.639 **	0.356 **	0.391 **
Plasma Se										1	0.820 **	0.593 **	0.263 **	0.535 **
Serum Se											1	0.651 **	0.374 **	0.626 **
Whole Blood GSH-Px												1	0.386 **	0.572 **
Plasma GSH-Px													1	0.763 **
Serum GSH-Px														1

Note: Asterisk indicates a statistically significant correlation coefficient, ** *p* < 0.01.

## Data Availability

Data are contained within the article.
